# NOX4 promotes Kupffer cell inflammatory response via ROS-NLRP3 to aggravate liver inflammatory injury in acute liver injury

**DOI:** 10.18632/aging.204173

**Published:** 2022-07-13

**Authors:** Liping Zhai, Hongyan Pei, Yi Yang, Yu Zhu, Shuiliang Ruan

**Affiliations:** 1 The Second Affiliated Hospital of Jiaxing University, Zhejiang 314001, China; 2 Jilin Agricultural University, Changchun 130000, China

**Keywords:** NOX4, acute liver injury, ROS, Kupffer, inflammatory response

## Abstract

Aim: This work aimed to investigate the mechanism of NOX4 in promoting Kupffer cells (KCs) activation and tissue inflammatory response in acute liver injury.

Methods: Initially, the mouse KCs were cultured *in vitro*. Thereafter, the NOX4 overexpression plasmid was transfected into KCs to construct the overexpression cell line. Then, KCs inflammatory response was induced by LPS + Nigericin treatment. CCK-8 assay was performed to detect cell viability, flow cytometry (FCM) was conducted to measure cell apoptosis, enzyme-linked immunosorbent assay (ELISA) was performed to detect inflammatory factor levels in the culture medium, NLRP3 and ASC expression in cells was detected by immunofluorescence (IF) staining, and ROS expression was detected by the DCFH-DA probe. Furthermore, the expression levels of NLRP3, ASC and Caspase-1 proteins were detected by Western-Blot (WB) assay. Furthermore, cells were pre-treated with NOX inhibitor or NAC to suppress NOX4 expression or ROS production, aiming to further investigate the effect on KCs inflammatory response. In mouse experiments, the NOX4 knockdown mice and wild-type (WT) mice were adopted for carrying out experiments. The mouse model of ALI was constructed with LPS and D-GalN treatment. Thereafter, the changes in tissue samples were detected by H&E staining, NLRP3 expression was measured by histochemical staining, inflammatory factors in tissues were analyzed by ELISA, and the levels of NLRP3, ASC and Caspase-1 proteins in tissues were detected by WB assay.

Results: LPS induced KCs inflammatory response. NOX4 overexpression decreased the mouse viability and increased the apoptosis rate. The levels of inflammatory factors were up-regulated in the culture medium. In addition, ROS were activated, and the positive cell number increased. Moreover, NOX4 promoted NLRP3 activation and significantly increased the expression of NLRP3 and ASC. Pretreatment with NOX4 inhibitor or NAC antagonized the effects of NOX4 and suppressed the KCs inflammatory response. In the mouse model, NOX4 knockdown significantly suppressed the activation and inflammatory response of microglial cells in tissues, reducing the NLRP3 expression in tissues.

Conclusion: NOX4 activates the NLRP3 inflammasome via ROS to promote inflammatory response in KCs and the release of inflammatory factors, suppressing NOX4 can improve ALI in mice, and NOX4 is promising as a new target for ALI treatment.

## INTRODUCTION

Kupffer cells (KCs) are the innate immune cells in the liver and are unique macrophages [1]. Occupying over 80% of the total immune cells, KCs have important roles in eliminating the pathogenic microorganisms and lipopolysaccharide (LPS) in the body, and exert the massive phagocytic function in KCs in the case of infectious disease [2]. However, LPS can induce the excessive activation of KCs, while the activated KCs mediate the synthesis and release of numerous inflammatory factors and cytokines, thus further aggravating liver injury [3]. The activation of KCs is regulated by multiple signals, while MAPK is a critical signal for KCs activation [4], which exerts its function through activating the downstream NF-κB and ROS. Besides, the massive expression of ROS, iNOS, and inflammatory factors IL-6, IL-1β and TNF-α will lead to KCs activation and injury [5–6].

ROS are the important cytokines that promote KCs activation. The source of ROS is mainly associated with the action of NAPDH oxidase, while NOX4 is one of the important NAPDH oxidases and the major source of ROS *in vivo* [7]. As discovered in current studies, NOX4 is related to liver fibrosis, and the suppression of NOX4 can inhibit liver fibrosis, while acute liver injury (ALI) is the early pathological process of liver fibrosis [8]. At present, the role of NOX4 in liver injury has not been illustrated. Therefore, the present work aimed to further investigate the role of NOX4 in KCs activation by adopting the liver injury model.

## MATERIALS AND METHODS

### Cell culture

The mouse liver KCs (Procell Biotechnology Co., Ltd, Wuhan, China) were cultured with the RPMI-1640 complete medium that contained 10% FBS and incubated under 37ºC, 5% CO_2_ and saturated humidity conditions. After cells were cultured to logarithmic phase, they were divided into Con, L/N-Con, NOX4 and L/N-NOX4 groups. KCs activation in L/N group was induced by LPS (1 μg/ml) combined with Nigericin (10 μM) (Sigma, MA, USA), while cells in Con group were adopted for control. Cells in NOX4 group were transfected with the lentivirus-packaged pGL3-NOX4 overexpression plasmid (Jikai Gene Co., Ltd, Suzhou, China) to construct the NOX4 overexpression KCs. Cells in L/N-NOX4 group were induced by LPS and Nigericin for cell activation.

To explore the role of NOX4, we divided KCs into Con, L/N and L/N + GKT groups in the second experiment. Among them, GKT was pretreated with the NOX4 inhibitor GKT137831 (MCE, Shanghai, China) at the final concentration of 150 nm for 6 h, followed by combined cell induction by LPS (1 μg/ml) and Nigericin (10 μM).

ROS are the effector molecules of NOX4. In our third experiment, KCs were divided into Con, L/N and L/N + NAC groups. Among them, NAC was pretreated with the ROS inhibitor at a concentration of 5 μM, followed by combined cell induction by LPS (1 μg/ml) and Nigericin (10 μM).

### CCK-8

KCs were inoculated into the 96-well plates. The medium was replaced by the serum-free medium when cells grew to 80% density, and cells were also treated with LPS and Nigericin at the same time. Cell viability was detected at 6 and 12 h, respectively. Later, 100 μl serum-free medium was added into each well, and then 10 μl CCK-8 reagent (Beyotime Biotechnology Institute, Shanghai, China) was also added to co-culture cells for 4 h, and OD value was detected at 450 nm. Then, the cell viability was calculated.

### Flow cytometry (FCM)

KCs were inoculated into the 6-well plates and cultured overnight for adherence. When cells grew to 80% density, the medium was replaced. After LPS/Nigericin induction for 12 h, the suspension and adherent cells were collected, washed with pre-chilled PBS, and stained with Annexin V-FITC/PI in line with kit (BD, New Jersey, USA) instructions. Subsequently, the cell apoptosis level was detected with the flow cytometer, and results were expressed as the proportion of Annexin V-FITC(+) PI(+) cells.

### ELISA

In KCs experiments, the expression levels of inflammatory factors IL-1β, IL-6 and TNF-α in culture medium were detected. Briefly, KCs were inoculated into the 12-well plates and induced by LPS/Nigericin for 12 h. Afterwards, the culture medium was collected and centrifuged at 3000 g to collect the supernatant, thereafter, the inflammatory factor levels in supernatant were measured using the ELISA kit (Nanjing Jiancheng Institute of Bioengineering, Nanjing, China). Later, reagent was added for incubation according to the instructions and OD value was measured at 450 nm with the microplate reader (BioTek, VT USA). The results were expressed as ng/ml.

In mouse experiment, the levels of inflammatory factors in peripheral blood and liver tissues of mice were detected. Briefly, the tail venous blood was collected from mice and centrifuged to obtain the supernatant serum. After mice were sacrificed by carbon dioxide suffocation, the liver tissues were ground with liquid nitrogen. Then, 1.0 ml RIPA lysate was added to lyse cells on ice, followed by centrifugation at 10000g for 15 min. Later, the supernatant was collected for protein quantification in line with the specific kit instructions. Results were expressed as ng/ml.

### Immunofluorescence (IF)

The NLRP3 and ASC protein levels in KCs were detected. KCs growing on glass coverslips were stained, inoculated into the 6-well plates, and treated with LPS/Nigericin for 12 h. Later, the medium was removed. Afterwards, cells were washed with pre-chilled PBS thrice, treated with 4% formaldehyde for 0.5 h, and permeabilized with 0.2% Triton X-100 for 5 min. NLRP3 monoclonal antibody (Abcam, MA, USA) was diluted at 1:300, while ASC monoclonal antibody (Abcam, MA, USA) was diluted at 1:500, and both of them were later used to incubate cells at 4ºC. Then, cells were washed twice with PBS, incubated with fluorescence secondary antibody, mounted with 95% glycerin, and observed under the fluorescence microscope.

### DCFH-DA

ROS were detected using the DCFH-DA probe (Beyotime Biotechnology Co., Ltd, Shanghai, China). In brief, KCs were inoculated into the 12-well plates, cultured overnight, and induced with LPS/Nigericin for 12 h. Thereafter, the DCFH-DA probe was diluted with serum-free medium at 1:1000, and 1 ml DCFH-DA probe-containing medium was supplemented into each well to further incubate cells in the incubator for 30 min. Then, the medium was discarded, and cells were washed with serum-free medium twice. The cell staining level was observed under the fluorescence microscope. In addition, the absorbance (OD) value was detected with the fluorescence spectrophotometer.

### Western-blot (WB) assay

In cell experiment, the protein levels were detected as follows. KCs were induced with LPS/Nigericin for 12 h first, then the suspension cells and adherent cells were collected, and washed with pre-chilled PBS twice. Later, 1.0 ml NP-40 lysate (Beyotime Biotechnology Co., Ltd, Shanghai, China) was added to lyse cells for 30 min on ice. Then, 5x loading buffer mixed protein solution was added, and the mixture was boiled for 8 min. Afterwards, proteins were separated by SDS-PAGE and transferred onto the PVDF membranes for 0.5–2 h. Later, the membranes were blocked with 5% skimmed milk powder for 2 h. The levels of NLRP3, ASC and Caspase-1 proteins were detected. The monoclonal antibody was diluted with TBST at 1:500, and added to incubate the membranes overnight 4ºC. Then, membranes were further incubated with HRP-IgG (Abcam, USA), the protein blots were detected with chemiluminescence (ECL), and the OD value were analyzed by Image Pro-Plus 6.0 software. Results were denoted as the OD ratio of target protein to endogenous reference protein.

Detection of proteins in liver tissues. Briefly, 100 mg liver tissues were minced with the sterile surgical scissors, grinded with liquid nitrogen, and lysed with 1.0 ml NP-40 lysate (Beyotime Biotechnology Co., Ltd, Shanghai, China) for 30 min on ice. Then, the sample was centrifuged at 10000 g for 15 min to collect the supernatant for protein quantification. The relative expression levels of proteins were detected in line with the above-mentioned detection method.

### Construction of the animal model

The female NOX4 knockdown mice (C57BL/6N-NOX4^em1cyagen^) and female WT C57BL/6 mice weighing 21–25 g were raised in the same environment. After adaptive feeding for 1 week, mice were divided into KO, WT, KO-D/L and WT-D/L groups. Among them, mice in KO and WT groups received routine feeding, whereas those in KO-D/L and WT-D/L groups received LPS and D-GalN treatment to construct the mouse model of liver injury. Mice in WT and KO groups were given intragastric administration of normal saline, whereas those in KO-D/L and WT-D/L groups were treated with 1000 mg/kg D-GalN (Sigma, MA, USA) and 10 μg/kg LPS (Sigma, MA, USA) via intraperitoneal injection to construct the ALI model, All mice survived.

### Histochemical staining

After injection with LPS/D-GalN for 72 h, mice were sacrificed with carbon dioxide suffocation to dissect the liver tissues. The liver tissues were embedded in paraffin, deparaffinized in xylene, and immersed with gradient ethanol, followed by antigen retrieval. Later, endogenous peroxidase was eliminated with 3% hydrogen peroxide. Next, sections were blocked with 2% bovine serum albumin (BSA) at 37ºC for 30 min to detect the NLRP3 expression. The NLRP3 monoclonal antibody was diluted at 1:350 (Abcam, USA). Later, the sections were incubated with peroxidase-labeled streptomycin (Abcam, USA) for 15 min, treated with the freshly prepared DAB solution for color developing (DAKO, Denmark), sufficiently washed with deionized water, counter-stained with hematoxylin, and mounted. All sections were photographed with the Olympus-BX51 upright metallurgical microscope equipped with the Olympus-DP72 image acquisition system and the CRi Nauance multispectral imaging system (Cambridge Research and Instrumentation, MA, USA).

### H&E

Mice were injected with LPS/D-GalN, and, 72 h later they were sacrificed through carbon dioxide suffocation, and the liver tissues were dissected. Liver tissues were later embedded in paraffin, and sliced into the 4-μm consecutive sections, followed by the staining steps below, xylene deparaffinage, gradient dehydration with ethanol (100%, 95%, 80%), washing with tap water for 2 min, staining with hematoxylin for 3 min. After washing with tap water for 2 min, sections were treated with 1% hydrochloric acid alcohol for 2 s, washed with tap water for 2 min, treated with 1% ammonia water for 20 s, stained with 0.5% eosin alcohol for 10 s, dehydrated with gradient alcohol, permeabilized with xylene, and mounted with neutral resin. Finally, the pathological changes of liver tissues were observed under the light microscope.

### Detection of AST and ALT

After intervention with LPS/D-GalN for 72 h, the tail venous blood was collected from each mouse, and centrifuged to collect the supernatant. ALT and AST levels were detected by ultraviolet colorimetry (Nanjing Jiancheng Institute of Bioengineering, Nanjing, China) in line with kit instructions. The results of AST and ALT were expressed as U/L.

### Statistical analysis

Measurement data were expressed as mean ± standard deviation ($\bar{x}$ ± s). Comparisons among multiple groups were conducted by one-way ANOVA, while comparisons between two groups were performed by two independent sample *t*-test. The above tests were all two-sided, and *P* < 0.05 stood for statistical significance.

### Data availability statement

The data that support the findings of this study are available from the corresponding author upon reasonable request.

## RESULTS

### NOX4 promoted KCs inflammatory response

**Figure 1:**
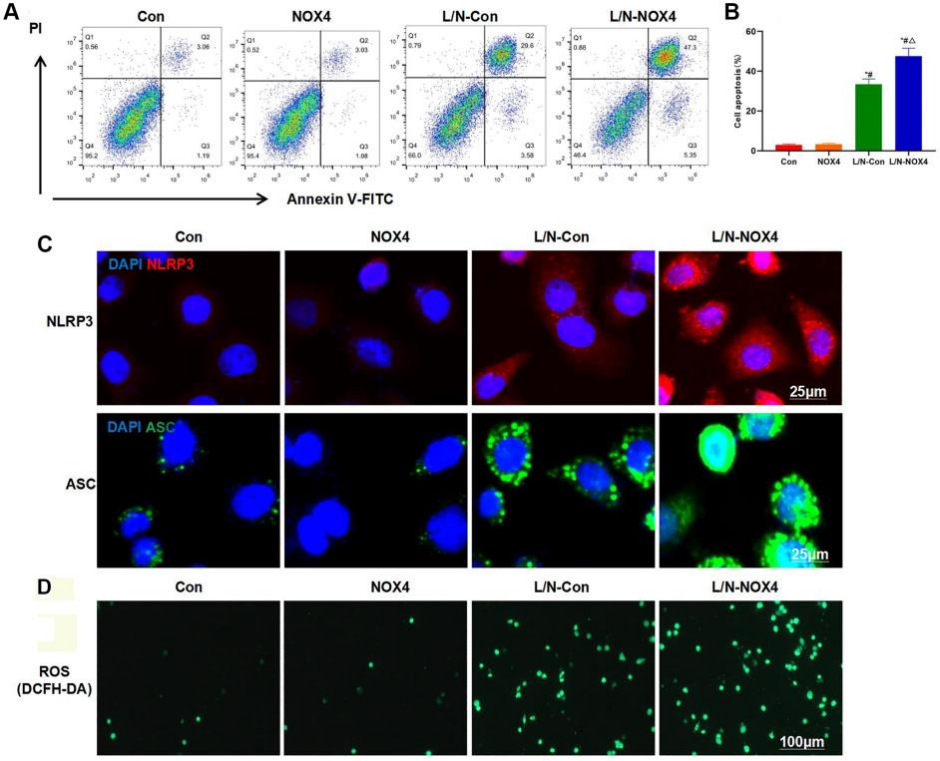
**NOX4 promotes inflammatory injury in KCs.** (**A**, **B**) Results of FCM assay (*n* = 3). The cell apoptosis levels in Con and NOX4 groups were relatively low, with no obvious inflammatory injury. Obvious cell apoptosis was observed in L/N-Con group, and the apoptosis rate was higher than that in Con and NOX4 groups. The cell apoptosis rate in L/N-Con group was further up-regulated, higher than that in L/N-Con group. ^*^P < 0.05, compared with Con group; ^#^*P* < 0.05, compared with NOX4 group; ^△^*P* < 0.05, compared with L/N-Con group. (**C**) Results of IF staining (*n* = 3). L/N promoted the activation of NLRP3 inflammasome, and the levels of NLRP3 and ASC increased. The levels NLRP3 and ASC further increased in L/N-NOX4 group, higher than those in L/N-Con group. (**D**) ROS detection by the DCFH-DA probe (*n* = 3). The numbers of positive cells in Con and NOX4 groups decreased, that in L/N group increased, and that in L/N-NOX4 group further increased, indicating that NOX4 promoted ROS expression under the inflammatory condition.

LPS and Nigericin induced inflammatory response and inflammatory injury in KCs. In the absence of intervention, NOX4 overexpression did not cause injury to KCs. The cell injury level in L/N-NOX4 group was significantly higher than that in L/N-Con group, indicating that NOX4 promoted KCs injury under inflammatory condition (Figure 1A, 1B). In IF staining, L/N promoted the activation of NLRP3 inflammasome, and up-regulated the levels of NLRP3 and ASC. Apart from that, NLRP3 and ASC levels further increased in L/N-NOX4 group, which were higher than those in L/N-Con group (Figure 1C). In ROS detection, ROS expression decreased in Con and NOX4 groups, while L/N promoted ROS expression, and the number of positive cells increased. The number of positive cells in L/N-NOX4 group further increased, indicating that NOX4 promoted ROS expression under inflammatory condition (Figure 1D).

**Figure 2:**
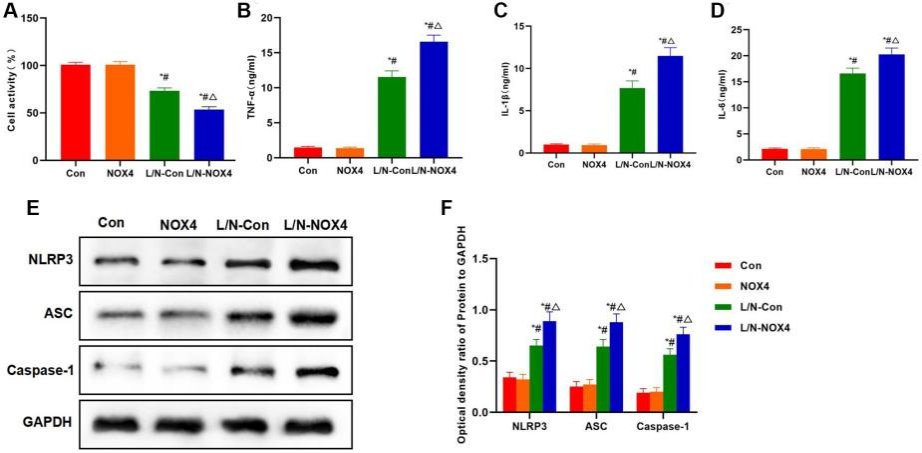
**NOX4 promotes inflammatory factor expression and NLRP3 inflammasome activation.** (**A**) Results of cell viability assay (*n* = 3). NOX4 promoted the down-regulation of cell viability. The cell viability in L/N-NOX4 group significantly decreased compared with L/N-Con group. ^*^P < 0.05, compared with Con group; ^#^*P* < 0.05, compared with NOX4 group; ^△^*P* < 0.05, compared with L/N-Con group. (**B**–**D**) Expression of inflammatory factors (*n* = 3). LPS and Nigericin promoted the expression and release of inflammatory factors, and the levels of IL-6, TNF-α and IL-1β were significantly higher than those in Con and NOX4 groups. While the expression of inflammatory factors in L/N-NOX4 group further increased, higher than that in L/N-Con group. ^*^P < 0.05, compared with Con group; ^#^*P* < 0.05, compared with NOX4 group; ^△^*P* < 0.05, compared with L/N-Con group. (**E**, **F**) Detection of protein expression (*n* = 3). LPS and Nigericin promoted NLRP3 activation, and the levels of NLRP3, ASC and Caspase-1 significantly increased. NOX4 overexpression further promoted NLRP3 activation, and the levels of NLRP3, ASC and Caspase-1 in L/N-NOX4 group were higher than those in L/N-Con group. ^*^P < 0.05, compared with Con group; ^#^*P* < 0.05, compared with NOX4 group; ^△^*P* < 0.05, compared with L/N-Con group.

Results of cell viability assay also indicated that, NOX4 promoted the down-regulation of cell viability under inflammatory condition, and the cell viability of L/N-NOX4 group was significantly lower than that of L/N-Con group (Figure 2A). As discovered from inflammatory factor detection, LPS and Nigericin promoted the expression and release of inflammatory factors, and the levels of IL-6, TNF-α and IL-1β were significantly higher than those in Con and NOX4 groups. While the expression levels of inflammatory factors in L/N-NOX4 group were further up-regulated, which were higher than those in L/N-Con group (Figure 2B–2D). Protein detection results also suggested that, LPS and Nigericin promoted NLRP3 activation, and markedly up-regulated the levels of NLRP3, ASC and Caspase-1. NOX4 overexpression further promoted NLRP3 activation, and the levels of NLRP3, ASC and Caspase-1 in L/N-NOX4 group were higher than those in L/N-Con group (Figure 2E, 2F).

### Suppressing NOX4 inhibited inflammatory injury and the release of inflammatory factors in KCs

**Figure 3:**
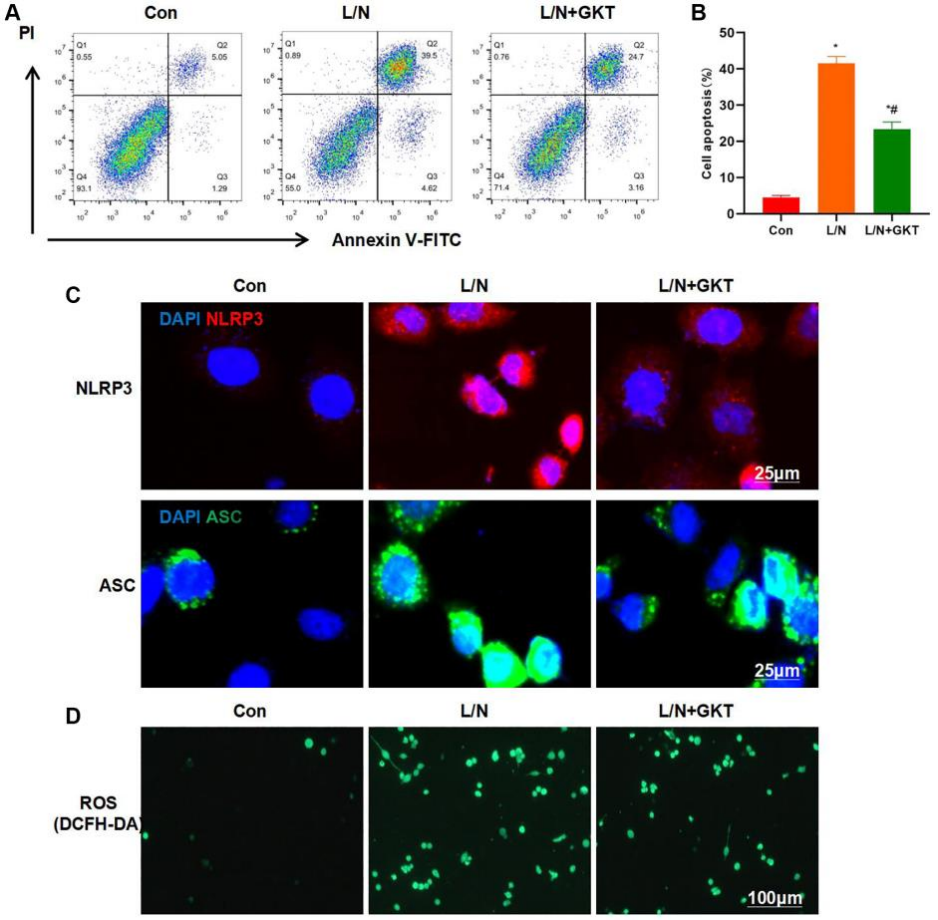
**Suppressing NOX4 inhibits inflammatory response and injury in KCs.** (**A**, **B**) Results of FCM assay (*n* = 3). The cell apoptosis level in L/N + GKT group significantly decreased, lower than that in L/N group. ^*^P < 0.05, compared with Con group; ^#^*P* < 0.05, compared with L/N group. (**C**) IF staining (*n* = 3). GKT suppressed NLRP3 inflammasome activation. The expression levels of NLRP3 and ASC evidently decreased, lower than those in L/N group. (**D**) ROS detection by DCFH-DA probe (*n* = 3). GKT inhibited ROS expression, and the number of positive cells markedly decreased, lower than that in L/N group.

GKT137831, the highly selective inhibitor of NOX4, was used for pretreatment. After GKT was used to suppress NOX4 expression, it resisted the LPS- and Nigericin-induced KCs injury. FCM results demonstrated that, the cell apoptosis level in L/N + GKT group significantly decreased, lower than that in L/N group (Figure 3A, 3B). Meanwhile, IF staining results suggested that, GKT suppressed the activation of NLRP3 inflammasome, and the expression levels of NLRP3 and ASC dramatically decreased, lower than those in L/N group (Figure 3C). As discovered after DCFH-DA probe detection, GKT suppressed ROS expression, and the number of positive cells significantly decreased, lower than that in L/N group (Figure 3D).

**Figure 4:**
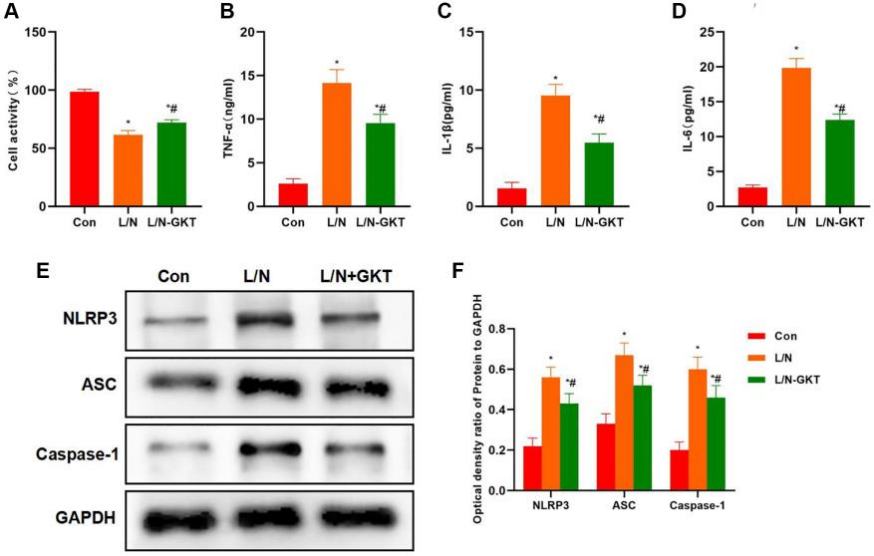
**Suppressing NOX4 inhibits inflammatory factor expression and NLRP3 activation.** (**A**) Results of cell viability assay (*n* = 3). GKT improved the cell viability, which was higher than that in L/N group. ^*^P < 0.05, compared with Con group; ^#^*P* < 0.05, compared with L/N group. (**B**–**D**) Expression of inflammatory factors (*n* = 3). GKT reduced the expression of inflammatory factors. The levels of IL-6, TNF-α and IL-1β in L/N + GKT group markedly decreased, lower than those in L/N group. ^*^P < 0.05, compared with Con group; ^#^*P* < 0.05, compared with L/N group. (**E**, **F**) Detection of protein expression (*n* = 3). After GKT suppressed NOX4, the activation of NLRP3 inflammasome was suppressed, and the expression of NLRP3, ASC and Caspase-1 decreased, significantly lower than that in L/N group. ^*^P < 0.05, compared with Con group; ^#^*P* < 0.05, compared with L/N group.

According to cell viability assay, GKT improved cell viability, which was higher than that in L/N group (Figure 4A). At the same time, GKT decreased the expression of inflammatory factors, and the levels of IL-6, TNF-α and IL-1β in L/N + GKT group remarkably decreased, lower than those in L/N group (Figure 4B–4D). Protein detection also discovered that, after GKT suppressed NOX4, the activation of NLRP3 inflammasome was suppressed, and the expression levels of NLRP3, ASC and Caspase-1 decreased, significantly lower than those in L/N group (Figure 4E, 4F).

### Suppressing ROS inhibited inflammatory injury and the release of inflammatory factors in KCs

**Figure 5:**
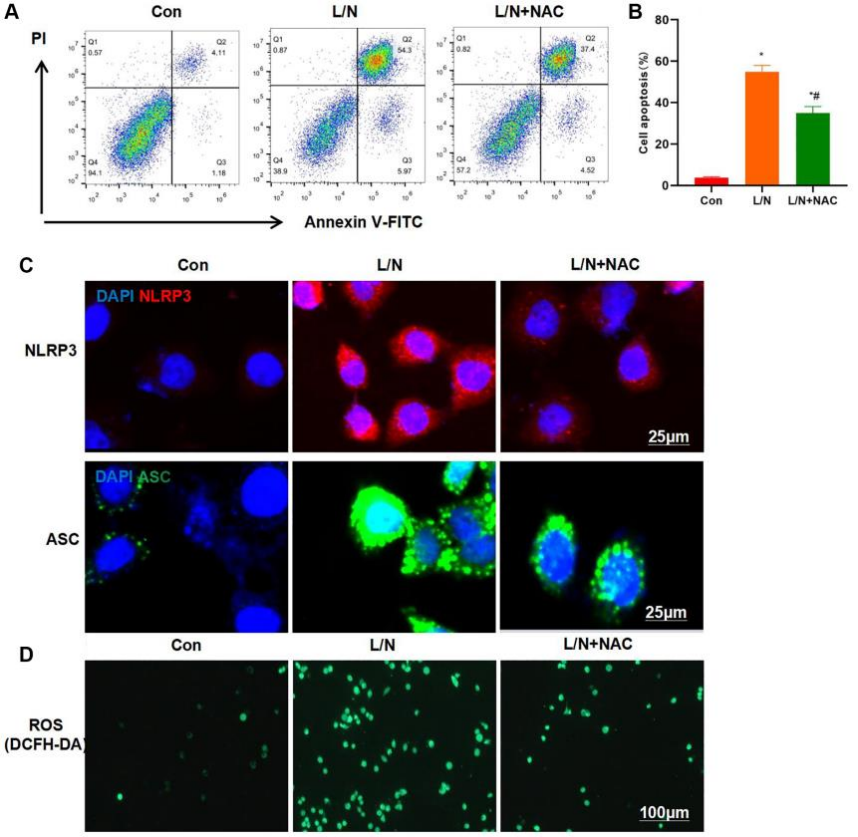
**Suppressing ROS inhibits inflammatory response and injury in KCs.** (**A**, **B**) Results of FCM assay (*n* = 3). The cell apoptosis level in L/N + NAC group was significantly down-regulated, lower than that in L/N group. ^*^P < 0.05, compared with Con group; ^#^*P* < 0.05, compared with L/N group. (**C**) IF staining results (*n* = 3). NAC suppressed the activation of NLRP3 inflammasome, and the expression levels of NLRP3 and ASC markedly decreased, lower than those in L/N group. (**D**) ROS detection by DCFH-DA probe (*n* = 3). NAC suppressed ROS expression, and the number of positive cells evidently decreased, lower than that in L/N group.

ROS are generated by NOX4. Therefore, cells were pretreated with the ROS inhibitor NAC in the current work. As a result, NAC also suppressed the inflammatory injury in KCs. Moreover, NAC resisted the LPS- and Nigericin-induced KCs injury. As revealed by FCM assay, the cell apoptosis level in L/N + NAC group significantly decreased, lower than that in L/N group (Figure 5A, 5B). Similarly, IF staining results also revealed that, NAC inhibited the activation of NLRP3 inflammasome, and the expression levels of NLRP3 and ASC were markedly down-regulated, lower than those in L/N group (Figure 5C). It was discovered after detection with DCFH-DA probe that, NAC suppressed ROS expression, and the number of positive cells significantly declined, which was lower than that in L/N group (Figure 5D).

**Figure 6:**
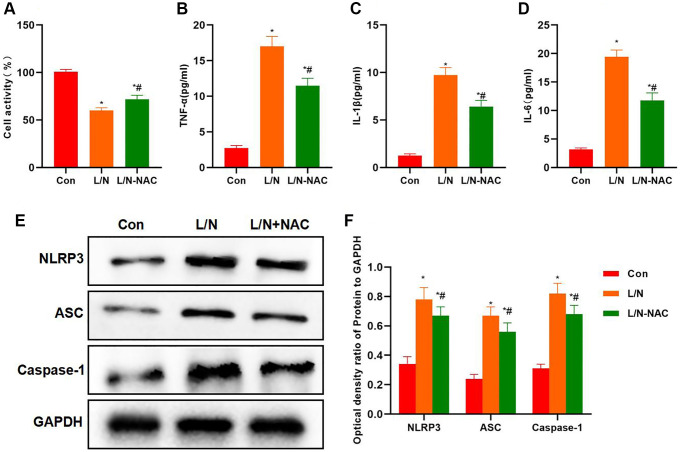
**Suppressing ROS inhibits inflammatory factor expression and NLRP3 activation.** (**A**) Results of cell viability assay (n-3). NAC improved cell viability, which was higher than that in L/N group. ^*^P < 0.05, compared with Con group; ^#^*P* < 0.05, compared with L/N group. (**B**–**D**) Expression of inflammatory factors (*n* = 3). NAC down-regulated the expression of inflammatory factors. The levels of IL-6, TNF-α and IL-1β in L/N + NAC group dramatically decreased, lower than those of L/N group. ^*^P < 0.05, compared with Con group; ^#^*P* < 0.05, compared with L/N group. (**E**, **F**) Detection of protein expression (*n* = 3). After NAC suppressed ROS, the activation of NLRP3 inflammasome was suppressed, the expression of NLRP3, ASC and Caspase-1 decreased, and the protein levels were dramatically lower than those in L/N group. ^*^P < 0.05, compared with Con group; ^#^*P* < 0.05, compared with L/N group.

Cell viability assay found that NAC improved cell viability, which was higher than that in L/N group (Figure 6A). Meanwhile, NAC reduced the expression of inflammatory factors, and the levels of IL-6, TNF-α and IL-1β in L/N + NAC evidently declined, lower than those in L/N group (Figure 6B–6D). Protein detection also suggested that, NAC suppressed the activation of NLRP3 inflammasome, and reduced the expression of NLRP3, ASC and Caspase-1, which was significantly lower than that in L/N group (Figure 6E, 6F).

### NOX4-KO alleviated inflammatory response in ALI tissues

**Figure 7:**
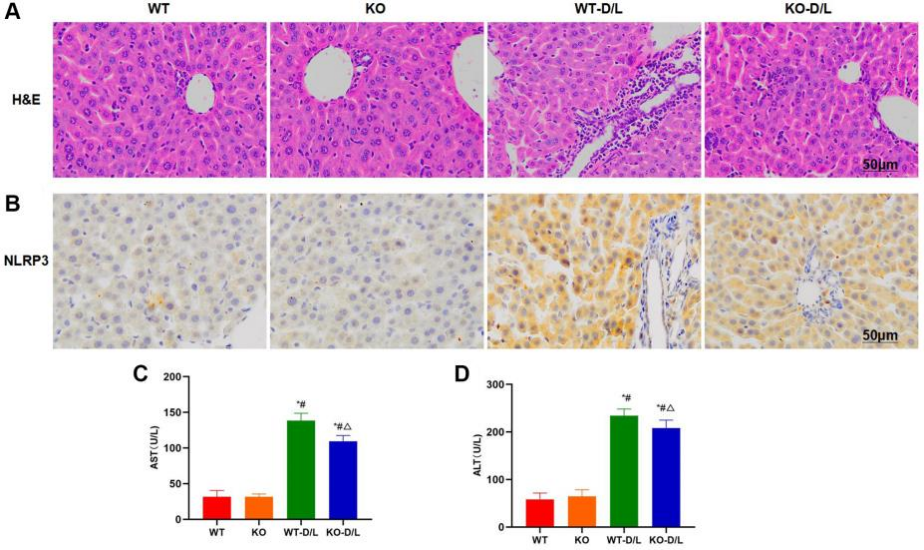
**NOX4-KO suppresses liver injury in mice.** (**A**) H&E staining (*n* = 5). Mice in KO and WT groups did not exhibit any obvious tissue injury, with no bubble-like structure or inflammatory response. Mice in WT-D/L group exhibited distinct inflammatory injury and obvious cell injury, along with inflammatory response. While tissue injury in KO-D/L group was alleviated. (**B**) Histochemical staining of NLRP3 (*n* = 5). NLRP3 expression dramatically increased in WT-D/L group, higher than that in WT and KO groups, while NLRP3 expression in WT and KO groups was relatively low, and that in KO-D/L group decreased. (**C**, **D**) Detection of ALT and AST levels (*n* = 5). ALT and AST levels dramatically decreased in KO-D/L group, lower than those in WT-D/L group. ^*^P < 0.05, compared with WT group; ^#^*P* < 0.05, compared with KO group; ^△^*P* < 0.05, compared with WT-D/L group.

We discovered in WT and KO mice that, after NOX4-KO treatment, the ALI degree in mice markedly mitigated, and the inflammatory response was suppressed. H&E staining results indicated that, mice in KO and WT groups did not exhibit any obvious injury, with no bubble-like structure or inflammatory response. Mice in WT-D/L group showed distinct inflammatory injury, with significant cell injury and inflammatory response, while tissue injury was mitigated in KO-D/L group (Figure 7A). Histochemical staining results indicated that NLRP3 expression significantly increased in WT-D/L group, higher than that in WT and KO groups, while NLRP3 expression was relatively low in WT and KO groups, and that in KO-D/L group decreased (Figure 7B). According to ALT and AST detection, ALT and AST levels significantly decreased in KO-D/L group, lower than those in WT-D/L group (Figure 7C, 7D).

**Figure 8:**
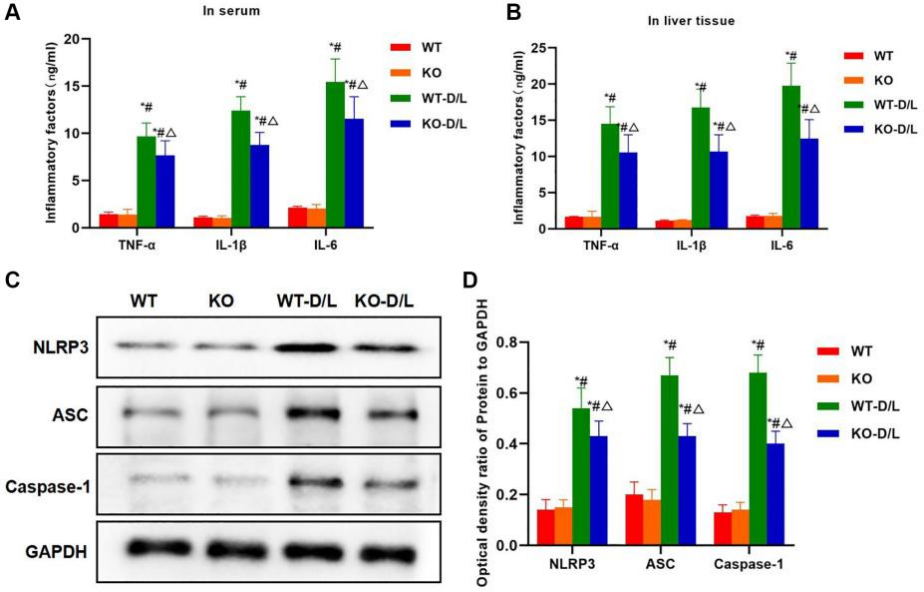
**NOX4-KO decreases inflammatory factor expression and NLRP3 inflammasome activation.** (**A**, **B**) Expression of inflammatory factors in serum and tissues (*n* = 5). The levels of IL-6, TNF-α and IL-1β in serum and liver tissues of WT-D/L group, higher than those in KO and WT groups while the differences between KO and WT groups were not significant. The inflammatory factor levels in tissues and serum of KO-D/L group declined, lower than those of WT-D/L group. ^*^P < 0.05, compared with WT group; ^#^*P* < 0.05, compared with KO group; ^△^*P* < 0.05, compared with WT-D/L group. (**C**, **D**) Detection of protein expression (*n* = 3). NLRP3 was activated in WT-D/L group, and the levels of NLRP3, ASC and Caspase-1 were dramatically higher than those in WT and KO groups. The protein levels in KO-D/L group decreased, lower than those in WT-D/L group. ^*^P < 0.05, compared with WT group; ^#^*P* < 0.05, compared with KO group; ^△^*P* < 0.05, compared with WT-D/L group.

Inflammatory factors were detected in serum and tissues. As a result, the levels of IL-6, TNF-α and IL-1β in serum and liver tissues of WT-D/L group evidently elevated, higher than those in KO and WT groups while the differences were not significant between KO and WT groups. The levels of inflammatory factors in tissues and serum of KO-D/L group decreased, lower than those in WT-D/L group (Figure 8A, 8B). Protein expression results demonstrated that, NLRP3 in WT-D/L group was activated, and the levels of NLRP3, ASC and Caspase-1 were remarkably higher than those in WT and KO groups, while the protein levels decreased in KO-D/L group, lower than those in WT-D/L group (Figure 8C, 8D).

## DISCUSSION

ALI is a common liver disease, inflammatory response in liver injury is an important factor that promotes tissue injury, while KCs activation exerts an important effect [9]. Research suggests that the number of KCs significantly increases in liver injury. KCs are a special type of macrophages [10], while the latter represents an important type of immune cells. KCs can be activated by interferon-γ (IFN-γ), tumor necrosis factor (TNF)-α and granulocyte-macrophage colony stimulating factor (GM-CSF) [11–12]. Under the liver injury status, KCs can recognize pathogenic microorganisms and secrete the inflammatory cytokines IL-1α, IL-1β and TNF-β, thus aggravating inflammatory response [13]. During this process, NLRP3 inflammasome is an important factor that mediates inflammatory factor maturation and expression [14–15]. Many studies have discovered that, NLRP3 is an important factor that promotes KCs activation [16–17], while NLRP3 activation is regulated by multiple signals, including ROS [18–19]. ROS can regulate the activation of NLRP3 inflammasome [20] and promote KCs polarization via NF-κB, while ROS also account for one of the major factors leading to liver injury [21]. NAPDH oxidase possesses four subtypes, namely, NOX1-4. Among them, NOX4 is extensively distributed and plays an important role in the mitochondrial respiratory chain electron transfer. Moreover, NOX4 is also one of the regulatory enzymes of ROS, which exerts a crucial function in multiple oxidative stress responses. NOX4 can produce massive ROS via catalytic oxidation, while ROS can promote the activation of NLRP3 and NF-κB, thus amplifying the inflammatory response.

As discovered in current research on liver injury, ROS exerts an important effect on liver injury. To be specific, ROS promotes liver injury progression and aggravates inflammatory response. Therefore, this study focused on the role of NOX4 in liver injury. To this end, KCs were used as the objects of study. First of all, NOX4 expression was over-expressed and cell inflammatory response was induced by LPS and Nigericin. The results suggested that NOX4 overexpression promoted inflammatory injury in KCs and markedly increased the apoptosis rate. In the meantime, NLRP3 inflammasome was activated, and the expression of NLRP3 and ASC was evidently up-regulated. Moreover, LPS and Nigericin induced ROS expression simultaneously. In NOX4 group, the number of ROS-positive cells apparently increased, indicating that NOX4 was one of the important sources of ROS in the ALI model. We discovered from inflammatory factor detection that, NOX4 promoted the expression of inflammatory factors, the levels of IL-6, IL-1β and TNF-α evidently increased, demonstrating that NOX4 was one of the key factors promoting inflammatory response. Protein detection results also suggested that, NOX4 promoted NLRP3 activation, which was one of the important mechanisms of ROS.

To further explore the role of NOX4, we treated cells with the NOX4 inhibitor GKT [21, 22]. As a result, after GKT suppressed NOX4 expression, the inflammatory response and injury in KCs decreased, and the apoptosis level and inflammatory factor levels were also reduced. Noteworthily, GKT suppressed NLRP3 activation and reduced ROS expression, further proving that NOX4 was one of the major sources of ROS in liver injury. As ROS was the effector of NOX4, this study suppressed ROS with NAC. The results suggested that ROS suppression also decreased NLRP3 inflammasome activation and the release of inflammatory factors. In animal models, we selected the NOX4-KO mice, and discovered that NLRP3 expression markedly decreased in the liver injury model after NOX4-KO treatment. At the same time, the liver injury degree was mitigated, inflammatory response and injury in tissues were suppressed, and the differences were significant compared with WT group. Further, the expression of inflammatory factors in peripheral blood and tissues of mice decreased. Therefore, we discovered at the cellular level and mouse level that, NOX4 was an important factor that promoted liver injury in mice.

## CONCLUSION

It is discovered in this study that NOX4 activates NLRP3 inflammasome via ROS, thus further promoting KCs inflammatory response. NOX4 suppression alleviates the ALI and tissue inflammatory response in mice. NOX4 is a potential therapeutic target for liver injury.
